# Finger Vein Recognition with Personalized Feature Selection

**DOI:** 10.3390/s130911243

**Published:** 2013-08-22

**Authors:** Xiaoming Xi, Gongping Yang, Yilong Yin, Xianjing Meng

**Affiliations:** School of Computer Science and Technology, Shandong University, Jinan 250101, China; E-Mails: fyzq10@126.com (X.X.); ylyin@sdu.edu.cn (Y.Y.); rongmengyuan@gmail.com (X.M.)

**Keywords:** finger vein recognition, feature extraction, PHGTOG, personalized feature selection

## Abstract

Finger veins are a promising biometric pattern for personalized identification in terms of their advantages over existing biometrics. Based on the spatial pyramid representation and the combination of more effective information such as gray, texture and shape, this paper proposes a simple but powerful feature, called Pyramid Histograms of Gray, Texture and Orientation Gradients (PHGTOG). For a finger vein image, PHGTOG can reflect the global spatial layout and local details of gray, texture and shape. To further improve the recognition performance and reduce the computational complexity, we select a personalized subset of features from PHGTOG for each subject by using the sparse weight vector, which is trained by using LASSO and called PFS-PHGTOG. We conduct extensive experiments to demonstrate the promise of the PHGTOG and PFS-PHGTOG, experimental results on our databases show that PHGTOG outperforms the other existing features. Moreover, PFS-PHGTOG can further boost the performance in comparison with PHGTOG.

## 1. Introduction

In recent years, there has been considerable research in finger-vein recognition due to its advantages over existing biometrics. As a biometric identifier, finger veins have the following properties [1]: (1) non-contact; (2) live-body identification; (3) high security; and (4) small device size. Therefore, personalized identification with finger vein patterns has received lots of research interest [2–6]. Currently, several commercial products are available for civilian applications [7–9].

Finger vein recognition involves four main steps: image capture, pre-processing, feature extraction and matching. In the image capture step, an infrared LED light of 760–1,000 nm is able to pass through the skin of the finger while the hemoglobin in the vein can absorb the infrared light [10], and then the finger vein patterns are captured by an infrared LED and CCD camera. The pre-processing procedure consists of image enhancement, normalization, *etc.* Enhancement algorithms [11–14] are utilized to enhance the images for better performance. Because the pre-processing procedure is not the core point of this paper, a detailed description of these approaches is not provided.

Feature extraction is the critical step in the finger vein recognition process. The feature extracting methods can be classified into two categories based on the rules that determine how the blood vessel network will be segmented. For the first category, the finger vein network is segmented first, and then the geometric shape, topological structure or other information of the segmented blood vessel network are obtained [15–22]. However, due to low qualities of finger vein images and the limitations of the segmenting algorithms, the segmentation results are often unsatisfying [23], hence, the feature based on the segmented blood vessel network is less powerful. To solve this problem, another category of feature extracting methods [10,23–27] are proposed, where after the pre-processing, the features will be extracted without segmentation. Although promising experiment results are reported in [22–27], two limitations of these feature extraction methods may exist. One problem is that the effective information contained in these features is not enough to make the feature more powerful and robust. For example, [24] proposes a LLBP operator for finger-vein recognition. The operator consists of two components: horizontal and vertical. These operators may lose other directional information, which may be important for recognition. In [10] PCA is proposed to extract the global features based on the rule of minimizing the image reconstruction errors. However, the global features may ignore some local detailed information, which is important for recognition. In addition, another limitation of these methods is that they treat all the features as equally important in the final matching. In fact, for a subject, only a subset of features distinguish this subject from other subjects. Moreover, the discriminative subset of features is different for different subjects due to the differences between the subjects. For example, [25] uses the LBP operator to extract the binary code for each subject, where every binary bit is treated as of equal importance. In fact, for a subject, only a part of the binary bit can really reflect the main characteristics of this subject and the discriminative bits are different for different subjects. The authors of [26] propose PBBM, which is rooted in LBP, to select the personalized discriminative bits for each subject. Experimental results show that PBBM achieves quality performance not only on the recognition accuracy but also in time cost due to the fact PBBM only selects a part of personalized discriminative bits for each subject. Although PBBM can improve the performance, PBBM based on LBP cannot achieve a satisfying result due to the insufficient information in the LBP. For example, LBP cannot reflect the delicate difference among the gray values of pixels. Given a pixel whose gray value is 100, the gray values of its eight neighbors are all 90, and its LBP code is 1111111, for another pixel whose gray value is 50, its eight neighbors are all 20, and its LBP code is 1111111. Although the LBP codes of the two pixels are same, the local gray differences of them are not same apparently.

As mentioned above, most of the proposed features only involve the shape information or texture information. To extract a more powerful feature, a combination of the effective information such as gray, texture and shape is necessary. In addition, the feature which reveals the global spatial layout and the local detail of the finger vein will make the feature more robust. In this paper, we propose a simple but powerful feature based on the spatial pyramid representation and a combination of more effective information such as gray, texture and shape. We construct the pyramid histogram of gray (PHG) and pyramid histogram of texture (PHT) by using a method which is similar to the method of extracting PHOG [28]. We join the pyramid histogram of gray, pyramid histogram of texture (LBP operators are used to form the pyramid histogram of texture) with PHOG [28] to reflect the global and local information of gray characteristic, texture characteristic and shape characteristic of the finger vein. We called our feature Pyramid Histograms of Gray, Texture and Orientation Gradients (PHGTOG). Although rich information will make the feature more robust, in fact, only a part of information is useful for distinguishing a subject from other subjects, moreover, the irrelevant information may degrade the recognition accuracy and increase the computational complexity. Therefore, selecting a subset of features containing the discriminative information is very important for the performance gain. In addition, another significant problem should not be ignored. That is the differences that exist between subjects. The discriminative subsets of features are different for different subjects. Therefore, it is crucial to select the personalized features for subjects. In this paper, the sparse weight vector for each subject is trained by using LASSO [29], we select a personalized subset of features from PHGTOG for each subject by using the sparse weight vector and we call the selected features PFS-PHGTOG.

The rest of this paper is organized as follows: we briefly introduce the PHGTOG extraction method in Section 2. Personalized feature selection will be introduced in Section 3. The proposed framework of finger vein recognition will be described in Section 4. Experimental results are demonstrated in Section 5 to verify the validity of the proposed approach. Finally, Section 6 concludes this paper.

## 2. PHGTOG for Image Representation

Based on spatial pyramid representation and a combination of more effective information such as gray, texture and shape, PHGTOG is designed as the Concatenation of the Pyramid Histogram of Gray (PHG), Pyramid Histogram of Texture (PHT) and PHOG [28]. Since spatial pyramid representation combines multiple resolutions in a principled fashion, it is robust to failures at the subject level [30]. PHG can represent the statistical information of global gray and local gray, PHT can reflect the statistical information of texture characteristics at multiple resolutions. PHOG can represent an image by its local shape and the spatial layout of the shape [28], so PHGTOG can represent the global spatial layout and local details of gray, texture and shape. We will describe the details of PHG, PHT and PHOG in the following subsection, PHGTOG is obtained by combining PHG, PHT and PHOG.

### 2.1. PHG Feature Extraction

The method of extracting PHG can be divided into three steps:

(a) Partition each image into a sequence of increasingly finer spatial grids by repeatedly doubling the number of divisions in each axis direction at each pyramid resolution level.
(b) Calculate gray histograms for each grid cell at each pyramid resolution level.
(c) Concatenate all the histogram vectors of all resolution levels to obtain the final feature for the image. The final feature is normalized to sum to unity.

**Figure 1.**
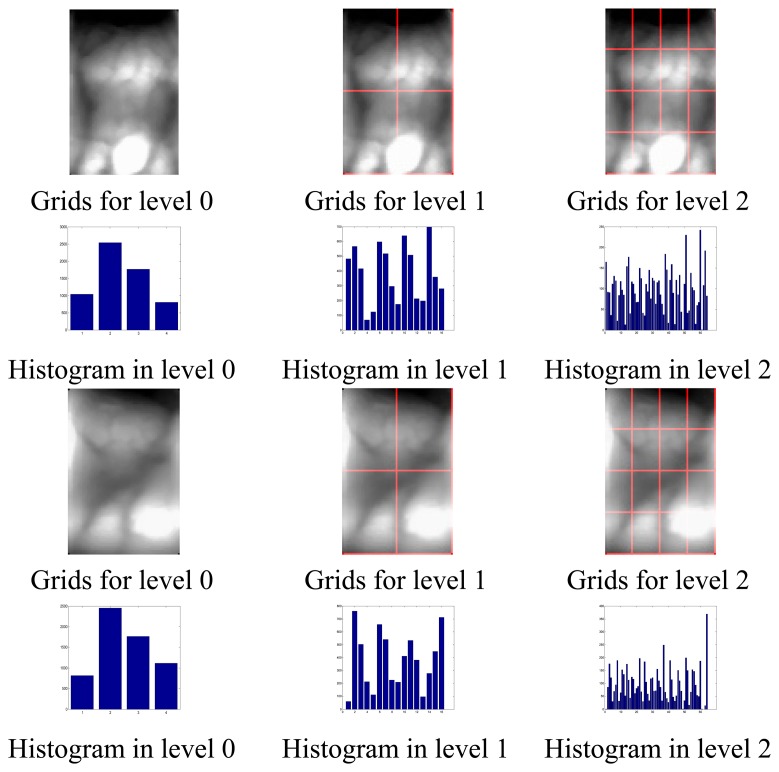
Gray spatial pyramid representation. The first row and third row: two images from different subjects and grids for levels L = 0 to L = 2; the second row and the fourth row: histogram representations corresponding to each level. The final PHG vector is a concatenation of vectors (histograms) for all levels.

Figure 1 gives out the results of gray spatial pyramid representation of two finger vein images (after pre-processing) from different subjects at different level resolution. As shown in Figure 1, level 0 is represented by a K-vector, where K (in Figure 1, K = 4) is the number of the bins of the histogram in each grid, level 1 by a 4K-vector, *etc.*, and the gray histogram of the entire image is a vector with dimensionality K. L is the pyramid resolution level of the image. Interestingly, the histogram in each grid can describe the local gray information, the gray feature in the level 0 can represent the global gray information of these two samples. Therefore, PHG can also reflect the global gray information to a certain degree.

From Figure 1 we can see that the feature extracted at the level 1 and 2 are more discriminative than that at the level 0 for the two images. In other words, the local features represent more detailed information, and thus are more discriminative than the global feature for the two images.

### 2.2. PHT Feature Extraction

The method of extracting the Pyramid histogram of Texture (the LBP operator [31] is used to represent texture) can be divided into three steps:

(a) Partition each image into a sequence of increasingly finer spatial grids by repeatedly doubling the number of divisions in each axis direction at each pyramid resolution level.
(b) Calculate LBP for the pixels in each grid cell at each pyramid resolution level. Based on the LBP code, each pixel can be represented by an 8 dimension vector whose values contain 0 and 1. The summation of all the 8 dimension vectors in the grid cell is the corresponding LBP histograms for this grid cell. For example, a grid cell contains four pixels. The LBP codes of the pixels are [0,1,1,1,0,1,0,0], [1,1,0,1,0,1,0,1], [0,1,1,1,0,1,0,1], [0,1,0,1,1,1,0,0], respectively. The LBP histogram of this grid cell is [1,4,2,4,1,4,0,2].
(c) Concatenate all the histogram vectors of all resolution levels to obtain the final feature for the image. The final feature is normalized to sum to unity.

**Figure 2.**
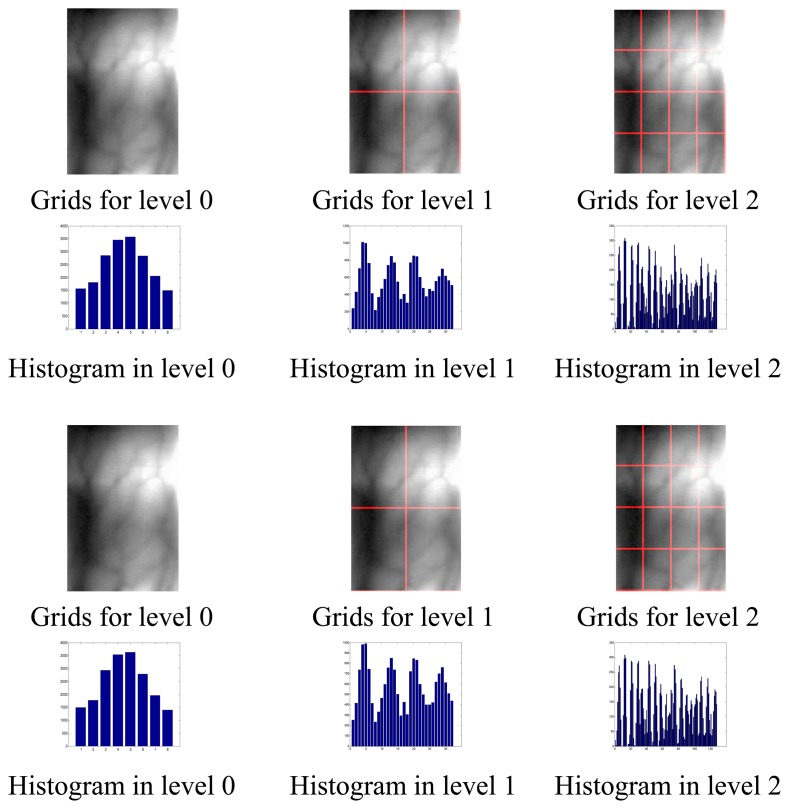
Texture spatial pyramid representation. The first row and the third row: two images from same subject and grids for levels L = 0 to L = 2; the second row and the fourth row: histogram representations corresponding to each level. The final PHT vector is a concatenation of vectors (histograms) for all levels.

Figure 2 gives the results of texture spatial pyramid representation of two finger vein images (after pre-processing) from the same subject at different level resolution. As shown in Figure 2, level 0 is represented by a K-vector corresponding to the K (K = 8) bins of the histogram, level 1 by a 4K-vector, *etc.*, and the texture histogram of the entire image is a vector with dimensionality K $\underset{l\in L}{\text{∑}}4^{l}$where *L* is the level of the image. The second finger vein image is captured by rotating the finger a small degree compared to the first image.

From Figure 2, although certain local texture features are different between these two samples due to the small rotation, the similar global texture characteristics will guarantee that the two images are classified into the same subject. We guess that the global texture feature may make PHT insensitive to the small rotation.

### 2.3. PHOG Feature Extraction

PHOG [28] is proposed as a spatial pyramid representation of the HOG descriptor. It achieved promising performance in object recognition, which can represent an image by its local shape and the spatial layout of the shape. We use PHOG to describe shape of the vein. Before extracting PHOG, the vein pattern should be firstly extracted by using the Sobel edge detector, and then we use the following three steps to obtain PHOG:

(a) Partition each image into a sequence of increasingly finer spatial grids by repeatedly doubling the number of divisions in each axis direction.
(b) Calculate Histograms of Orientation Gradients (HOG) [32] for each grid cell at each pyramid resolution level.
(c) The final PHOG descriptor for the image is a weighted concatenation of all the HOG vectors. The PHOG is normalized to sum to unity.

**Figure 3.**
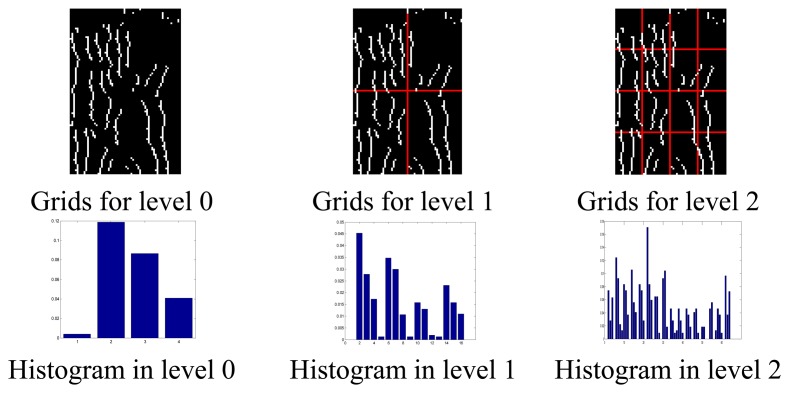
Shape spatial pyramid representation. The first row: an images and grids for levels L = 0 to L = 2; the second row: histogram representations corresponding to each level.

Figure 3 gives out the results of shape spatial pyramid representation of a finger vein image at different level. More details about PHOG can be found in [28]. Given that max(F) and min(F) are the maximum and minimum values of the raw features respectively, the normalized value of the feature is calculated as *f'* = [max(F) – *f*)/(*f* – min(F)]. In our experiments, the dimension of PHG, PHT, and PHOG are 84, 168, 340, respectively. We concatenate PHG, PHT and PHOG into a 592 dimension feature vector to obtain PHGTOG.

## 3. Personalized Feature Selection

After extracting the new feature PHGTOG, a high dimension feature vector can be obtained. Although PHGTOG contains a wealth of information, such a high dimension feature may contain some useless information which may degrade the recognition accuracy in the final matching, in addition, high dimension features may increase the computational complexity, so it can be very important to select a discriminative subset of PHGTOG for the final recognition.

Because the differences among subjects exist, personalized features that reflect the differences among subjects should be selected for different subjects. For example, subject 1 is distinguished from the other subjects by its shape characteristics, and subset of the feature that reflects the shape information of the subject 1 should be selected. The texture information makes the subject 2 very discriminative, and subset of the features that reflect the texture information are more important for subject 2 and should be selected for classifying subject 2 from other subjects.

In this paper, LASSO [29] was used to select the personalized feature for different subjects. It has frequently been observed that L1 regularization in many models causes many parameters to equal zero, so that the parameter vector is sparse[33].Linear least-squares regression with L1 regularization is called the LASSO [29] algorithm, which is known to generally give sparse weight vectors which contain some coefficients equals zero. The subset of the features which are useless for the matching can be ignored according to the weight coefficients which equals zero. We can get the sparse weight vector **w** by solving the optimization problem of minimizing following Equation (1):

(1) $$\text{Min}\sum_{i=1}^{m}(Y_{i}-w^{T}X_{i})+\lambda {\left\|w\right\|}_{1}$$

In Equation (1), we consider the training set *S* = { (*X_i_*,*Y_i_*) }, (I = 1,2,3,…*m*) with *m* training samples, where *Y_i_* ∈ {+1,−1 } is a class label, and *X_i_* is a p dimensional column vector which represents the feature of a sample. ***w*** = (*w*_1_,*w*_2_,…*w*_p_)^T^ is the weight coefficient vector, where *w_i_*(I = 1,2,…p) denotes the weight coefficient of the *i*-th dimension of the feature. Resulting from the difference among subjects, we should select the personalized subset of features for each subject, so we learn different ***w*** values for different subject. When we learn the weight coefficient vector of the *i*-th subject, the labels of the training samples from the *i*-th subject are +1, the remains are -1. After ***w*** is trained, we can select a subset of features from PHGTOG according to the weight vector ***w*** of each subject. We adopt the SLEP toolbox [34] to solve Equation (1). The selected features we called PFS-PHGTOG.

## 4. The Proposed Method

**Figure 4.**
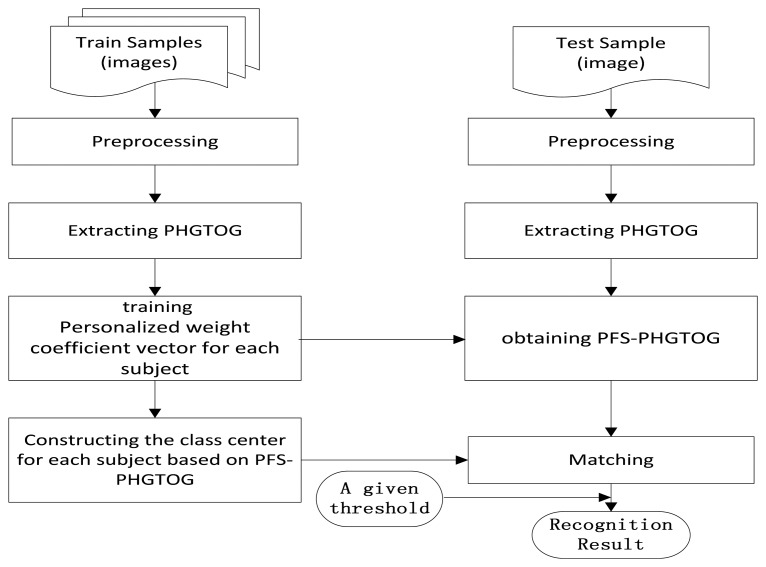
Framework of the proposed method.

In this section, we propose a finger vein recognition method based on PFS-PHGTOG. It mainly involves two stages: a training stage and a recognition stage. The training stage aims to train different weight coefficient vectors ***w*** for each subject and generate the class center based on PFS-PHGTOG for each subject. This stage includes preprocessing, PHGTOG extraction, personalized weight coefficient vector training, and we construct a class center for each subject based on PFS-PHGTOG. In the recognition stage, we first preprocess the test sample, and then extract PHGTOG, and get PFS-PHGTOG based on the above trained weight coefficient vector. Then the similarity between the test sample and the class center of a certain subject is computed, finally a recognition result is obtained by comparing the similarity with a given threshold. The framework of the proposed method is demonstrated in Figure 4.

### 4.1. Preprocessing

**Figure 5.**
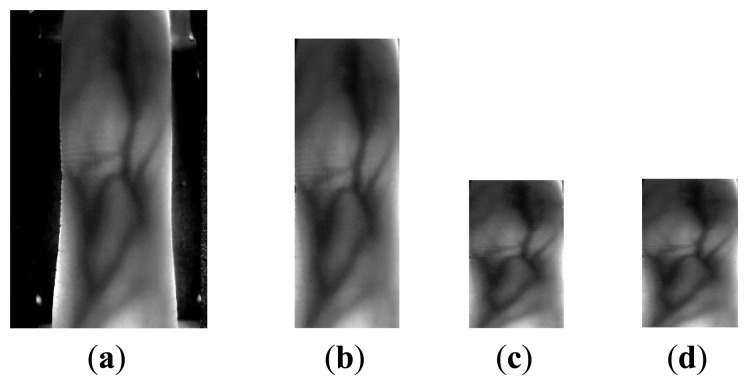
Examples of preprocessing. (**a**) Original finger vein image; (**b**) ROI of (a); (**c**) Finger vein image after size normalization; (**d**) Finger vein image after gray normalization.

For obtaining efficient features, image preprocessing is necessary. We use the preprocessing method proposed in [26] in this paper. The preprocessing mainly includes image gray processing, ROI extraction, size normalization and gray normalization. For reducing the computational complexity, image gray processing is used for transforming the original 24-bit color image (as shown in Figure 5a) into an 8-bit gray image. Edge-detection method with a Sobel operator can be used for extracting the finger contour. Region of Interest (ROI) (as shown in Figure 5b) can be obtained according to the maximum and minimum abscissa values of the finger contour. Due to personalized factors such as different finger size and changing location, the ROI region is normalized to the same size by using the bilinear interpolation, the size of the normalized ROI is set to be 96 × 64 (as shown in Figure 5c). After size normalization, gray normalization (as shown in Figure 5d) is used to obtain a uniform ray distribution.

### 4.2. Class Center Construction

We construct a class center for each subject with the training samples from this subject, where the *k*-th feature of the class center is calculated by averaging the corresponding feature values of the training samples. As there are N subjects, we then obtain N class centers *T_i_* (*i* = 1, 2, …, N).as shown in Equation (2):

(2) $$T_{i}=\frac{\sum_{j=1}^{M}q_{j}}{M}$$

In Equation (2), *q_j_* is the *j*-th sample represented by PFS-PHGTOG from the *i*-th subject. The number of the training samples in the *i*-th subject is M.

### 4.3. Matching

For any testing sample *f*, we estimate the similarity between this testing sample and the *i*-th class center *T_i_* with Euclidean distances as shown in Equation (3):

(3) $$D(f,T_{i})={\left\|f-T_{i}\right\|}_{2}$$

## 5. Experimental Results

### 5.1. The Experimental Database

**Figure 6.**
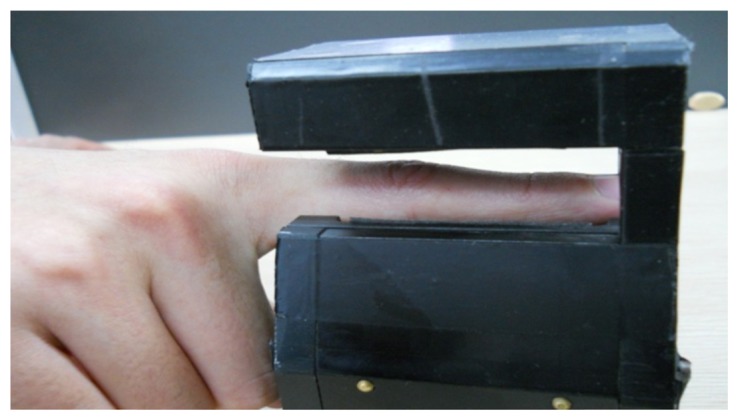
The finger vein image capture device.

**Figure 7.**
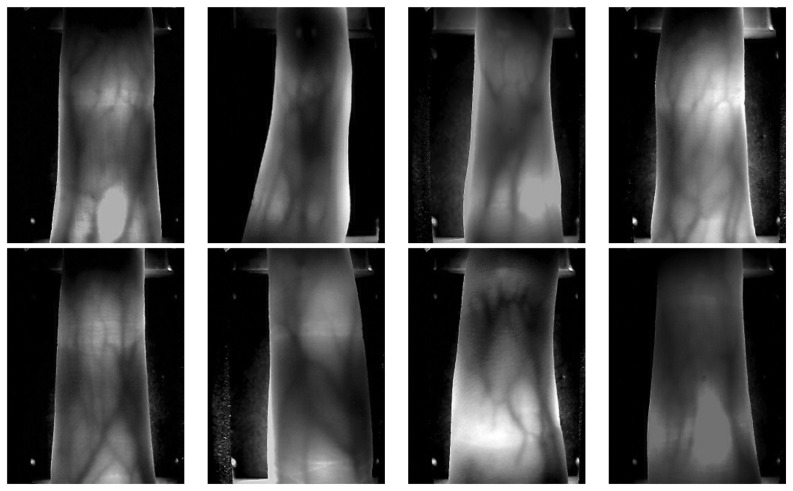
Sample finger vein images.

The experiments were conducted using our finger vein image database. Our finger vein image database consists of 4,080 images acquired from 34 volunteers (20 males and 14 females, Asian race) who are students, professors and staff at our school. To acquire these natural finger vein images. The finger vein images were acquired in two separate sessions with an interval of 20 days. The age of the volunteers was between 19 and 48 years. Each volunteer provides four fingers, which are left index, left middle, right index and right middle fingers, each of which contributes 30 images. In the first session, 20 images of each subject are captured, and the 10 images of each subject in the second session. We treat a finger as a subject. The capture device was manufactured by the Joint Lab for Intelligent Computing and Intelligent System of Wuhan University, China. The capture device mainly consists of a near-infrared light source, lens, light filter, and image capture equipment. Vein patterns can be viewed through an image sensor which is sensitive to near-infrared light (wavelengths between 700 and 1,000 nanometers), because near-infrared light passes through human body tissues and is blocked by pigments such as hemoglobin or melanin. To obtain a stable finger-vein pattern, our light source uses a near-infrared light source with wavelength of 790 nm. A groove in the shell of the device is used to guide the finger orientation, and the capture device is shown in Figure 6. The original spatial resolution of the image is 320 × 240. Several finger vein images in our database are shown in Figure 7.

Note that although [6] provides a public databases for finger vein images, the size of samples is too small for training the weight vector ***w*** in our work because the number of the finger vein images from each subject is 12.

### 5.2. The Experiment Settings

All the experiments are implemented in MATLAB 7.6, and run on a PC with a 2.9 GHz CPU and 4.0 GB memory. In the step of extracting PHGOTOG, the bins of the histogram in every grid cell is 4 (except for PHT, where the PHT bins are 8), the resolution level of PHG, PHT and PHOG are 2, 2 and 3, respectively. We conduct four experiments to demonstrate the efficiency of PHGTOG and PFS-PHGTOG: (1) Experiment 1 evaluates the efficiency of PHGTOG by comparing PHGTOG with PHG PHT and PHOG; (2) in Experiment 2, we compare the efficiency of PHGTOG with several traditional features such as mean curvature [17], LLBP [24], LBP, and LDP [25]; (3) in Experiment 3 we compare the efficiency of PHGTOG with PFS-PHGTOG to demonstrate the power of PFS-PHGTOG; (4) in Experiment 4, we evaluate the effect on recognition performance when using different numbers of training samples to train the weight coefficient vector for each subject.

### 5.3. Experiment 1

We performed this experiment on our finger vein database. Different fingers from the same individual were treated as different classes (*i.e.*, 136 classes), in the experiments, we use the first 10 samples of each class in the database to generate the class center and use the last 10 as test samples. Consequently, there are 1,360 (136 × 10) genuine ones. For obtaining the imposters, we select two samples from test samples of each class to match with the class center from different classes. So we can obtain 36,720 (135 × 2 × 136) imposters.

In this experiment, the performance of a system is evaluated by the equal error rate (EER), the false rejection rate (FRR) at zero false acceptance rate (FAR) and the FAR at zero FRR. EER is the error rate when the FRR equals the FAR and therefore suited for measuring the overall performance of biometrics systems because the FRR and FAR are treated equally. On the other hand, the FRR at-zero-FAR is suited for high security systems, as in those systems, false acceptance errors are much more critical than false rejection errors. On the contrary, the FAR at zero FRR shows the acceptance rate of impostors when none genuine rejected is desired.

**Figure 8.**
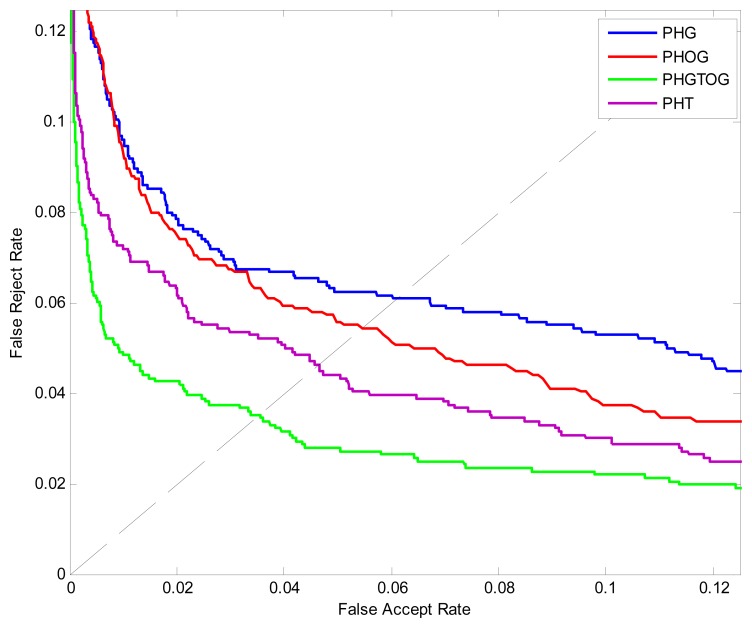
ROC curves of different features.

The ROC curves are shown in Figure 8. The ERR, FRR at-zero-FAR and FAR at-zero-FRR values are listed in Table 1. From Figure 8 and Table 1, we can see that PHGTOG achieves a much lower EER, FAR at-zero-FRR, FRR at-zero-FAR than PHG, PHT and PHOG. This can be explained that the combination of gray, texture, shape make PHGTOG contain more effective information and is more robust than the other two features which only reflect the shape or gray information.

### 5.4. Experiment 2

In this experiment, we compare the efficiency of PHGTOG with several traditional features extracted by the mean curvature [17], LLBP [24], LBP, and LDP [25] methods. The performance of different features is evaluated by the EER and recognition rate. In the identification mode, we want to identify input finger vein which class it belongs to. We use Equation (2) to construct the class center for every class, there are 136 class centers in total. A test image was matched with all the class centers according to Equation (3). If (f,T_j_) = min D(f,T_i_)(i = 1, 2, …, 136), then q goes to the *j*-th class. We can compare the prediction label and its truth label to judge if this test image is classified correctly. For N test images, we assume u test images are classified correctly, and then the recognition rate can be calculated as *u*/*N*.

**Figure 9.**
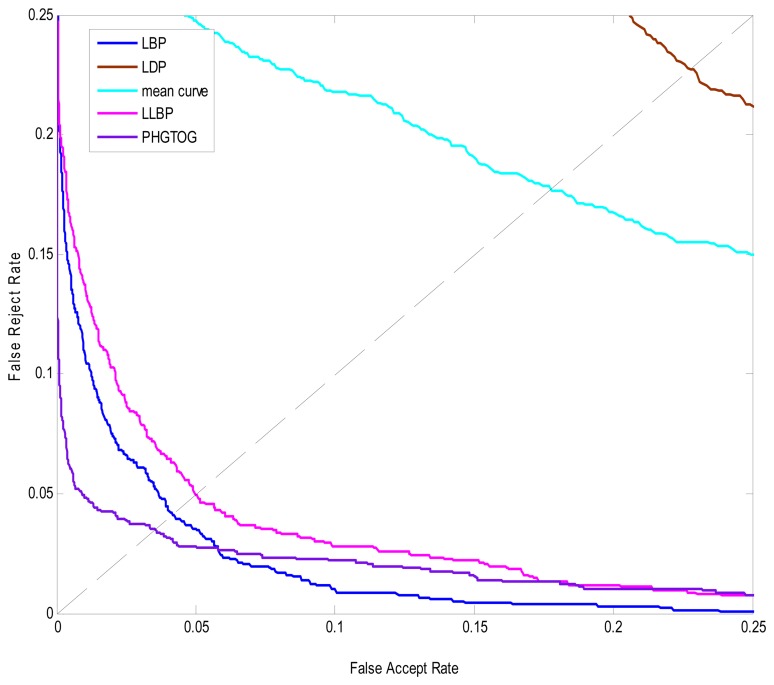
ROC curves of PHGTOG and different traditional features.

The ROC curves are shown in Figure 9. The ERR, recognition rate values are reported in Table 2. From Figure 9 and Table 2, we can see that PHGTOG achieves the best performance. Compared with the traditional features which only reflect one certain characteristic such as texture or shape, PHGTOG can describe not only global layout and local details, but also a combination of gray, texture and shape.

A wealth of information makes PHGOTG less insensitive to the noise and more discriminative than traditional features. Therefore, PHGOTG is more powerful and robust.

### 5.5. Experiment 3

**Figure 10.**
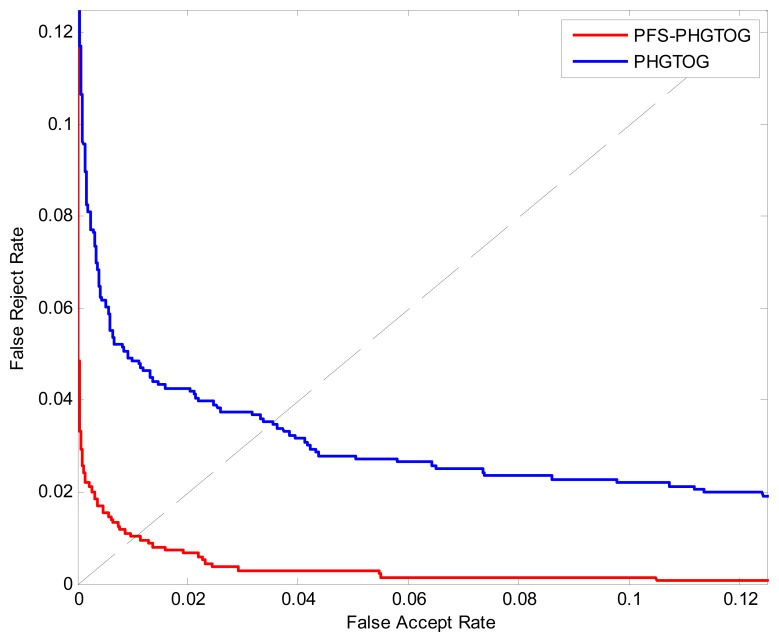
ROC curves of two different features.

In this experiment, we compare PHGTOG with PFS-PHGTOG to demonstrate the efficiency of personalized feature selection. The ROC curves of these two features are shown in Figure 10. The ERR, FRR at-zero-FAR and FAR at-zero-FRR values are listed in Table 3.

From Figure 10 and Table 3, we can see that PFS-PHGTOG achieves much lower EER, FRR at-zero-FAR and FAR at-zero-FRR. We think the reason may be that although PHGTOG contains rich information, only a subset of features can reflect the discriminative characteristics of the each subject, the discriminative subset of features is treated to be equally important as the other features which may be useless for matching, and this may limit the improvement of the performance. However, PFS-PHGTOG selects the distinguishing subset of features by considering the difference between the subjects, and the discriminative subset of features can play a more important role in the procedure of matching, so it results in a performance gain.

We also test the matching time to demonstrate that PFS-PHGTOG can reduce the computational complexity in comparison with PHGTOG. The matching time is shown in Table 4. Because PFS-PHGTOG is only a subset of PHGTOG, PFS-PHGTOG can achieve faster matching speed. Table 5 shows the maximum, minimum, average dimension of PHGTOG and PFS-PHGTOG of the subjects. As shown in Table 5, the dimension of most PFS-PHGTOG is much lower than PHGTOG.

### 5.6. Experiment 4

In this experiment, we evaluate the effect on the performance when using different numbers of training samples to generate PFS-PHGTOG.

For each subject, we select 8, 10, 12, 14, 16, 18, 20 samples from the first 20 images as the training samples respectively and the last 10 images as test samples. We generate genuine and imposters by the same methods as mentioned in Experiment 1.

We use EER and recognition rate as the performance measure. The EER, recognition rate values are listed in Table 6. From Table 6, we can see that the performance is improved with the number of training samples increased on the whole. The reason may be that prior knowledge is increased with the increased number of training samples, therefore, more prior knowledge makes the learned weight coefficient more optimal for feature selection, so the feature selected is more discriminative for each subject. Interestingly, the performance of PFS-PHGTOG with eight samples is much better than any other feature as shown in Experiment 2. The reason may be that although the number of the training samples is smaller, the effective information which PFS-PHGTOG represents is more and the crucial discriminative information is selected to play a key role for the recognition.

## 6. Conclusions and Future Work

In this paper, we firstly propose a very simple but powerful feature called PHGTOG which reflects not only the global spatial layout but also the local information of gray, texture and shape. Secondly, PFS-PHGTOG is proposed as a personalized subset of features from PHGTOG. Compared with PHGTOG, PFS-PHGTOG is not only more effective but also has a lower computational complexity. At last, we conduct experiments to demonstrate the efficiency of PHGTOG and PFS-PHGTOG.

As shown in Experiment 4, the performance is improved with sufficient training samples, but in some application areas, it is very difficult or expensive to obtain sufficient instances for training, so, finding a more powerful feature selection method to make full use of fewer samples to select the more effective feature will be the focus of our future work.

## Figures and Tables

**Table 1.** Performance of three different features.

		**EER**		**FAR at-zero-FRR**		**FRR at-zero-FAR**	
	PHG	1.	0.0610	2.	0.7995	3.	0.2699
4.	PHOG	5.	0.0545	6.	0.9320	7.	0.3493
8.	PHT	9.	0.0463	10.	0.6514	11.	0.2353
12.	PHGTOG	13.	0.0353	14.	0.5915	15.	0.1522

**Table 2.** Performance of PHGTOG and different traditional features.

	**EER**		**Recognition Rate**	
LBP	16.	0.0413	17.	0.9324
LDP	18.	0.2276	19.	0.6463
Mean Curve	20.	0.1775	21.	0.7007
LLBP	22.	0.0499	23.	0.9015
PHGTOG	24.	0.0353	25.	0.9765

**Table 3.** Performance of two different features.

	**EER**		**FAR at-Zero-FRR**		**FRR at-Zero-FAR**	
PHGTOG	26.	0.0353	27.	0.5915	28.	0.1522
29. PFS-PHGTOG	30.	0.0110	31.	0.3680	32.	0.0801

**Table 4.** Matching time of different features.

	**Average Time(ms)**
PHGTOG	1.5
PFS-PHGTOG	0.81

**Table 5.** Dimensions of PHGTOG and PFS-PHGTOG.

	**Maximum**		**Minimum**		**Average**	
PHGTOG	33.	592	34.	592	35.	592
PFS-PHGTOG	36.	351	37.	152	38.	302

**Table 6.** Performance with different numbers of training samples.

**Number of Training Samples**	**EER**		**Recognition Rate**	
8	39.	0.0103	40.	0.9603
10	41.	0.011	42.	0.9596
12	43.	0.0081	44.	0.9691
14	45.	0.0068	46.	0.9706
16	47.	0.0044	48.	0.9787
18	49.	0.0046	50.	0.9779
20	51.	0.0022	52.	0.9890

## References

[1] Liu Z., Yin Y.L., Wang H.J., Song S.L., Li Q.L. (2010). Finger vein recognition with manifold learning. J. Netw. Comput..

[2] Kono M., Ueki H., Umemura S. (2002). Near-infrared finger vein patterns for personal identification. Appl. Opt..

[3] Hashimoto J. Finger Vein Authentication Technology and Its Future.

[4] Mulyono D., Jinn H.S. A Study of Finger Vein Biometric for Personal Identification.

[5] Li S.Z., Jain A.K. (2009). Encyclopedia of Biometrics.

[6] Kumar A., Zhou Y. (2012). Human identification using finger images. IEEE Trans. Image Process..

[7] Finger Vein Products. http://www.hitachi.co.jp/products/it/veinid/global//products/index.html.

[8] Kiyomizu H., Miura N., Miyatakeand T., Nagasaka A. (2010). Finger Vein Authentication Device.

[9] Sato H. (2010). Finger Vein Authentication Apparatus and Finger Vein Authentication Method.

[10] Wu J.D., Liu C.T. (2011). Finger-vein pattern identification using principal component analysis and the neural network technique. Expert Syst. Appl..

[11] Yu C.-B., Zhang D.-M., Li H.-B. Finger Vein Image Enhancement Based on Multi-Threshold Fuzzy Algorithm.

[12] Yang J.F., Yang J.L. Multi-Channel Gabor Finger Design for Finger Vein Image Enhancement.

[13] Yang J.F., Yan M.F. An Improved Method for Finger-Vein Image Enhancement.

[14] Yang J.F., Yang J.L., Shi Y.H. (2009). Combination of Gabor Wavelets and Circular Gabor Filter for Finger-Vein Extraction.

[15] Miura N., Nagasaka A., Miyatake T. (2004). Feature extraction of finger-vein patterns based on repeated line tracking and its application to personal identification. Mach. Vis. Appl..

[16] Huafeng Q., Lan Q., Chengbo Y. (2011). Region growth-based feature extraction method for finger vein recognition. Opt. Eng..

[17] Song W., Kim T., Kim H.C., Choi J.H., Kong H.J., Lee S.R. (2011). A finger-vein verification system using mean curvature. Pattern Recognit. Lett..

[18] Yu C.B., Qin H.F., Zhang L., Cui Y.Z. (2009). Finger-vein image recognition combining modified Hausdorff distance with minutiae feature matching. J. Biomed. Sci..

[19] Hoshyar A.N., Sulaiman R., Houshyar A.N. (2011). Smart access control with finger vein authentication and neural network. J. Am. Sci..

[20] Huang B.N., Dai Y.G., Li R.F. Finger-Vein Authentication Based on Wide Line Detector and Pattern Normalization.

[21] Miura N., Nagasaka A., Miyatake T. Extraction of Finger-Vein Patterns Using Maximum Curvature Points in Image Profiles.

[22] Zhang Z., Ma S., Han X. Multiscale Feature Extraction of Finger-Vein Patterns Based on Curvelets and Local Interconnection Structure Neural Network.

[23] Wu J.D., Ye S.H. (2009). Driver identification using finger-vein patterns with Radon transform and neural network. Expert Syst. Appl..

[24] Rosdi B.A., Shing C.W., Suandi S.A. (2010). Finger vein recognition using local line binary pattern. Sensors.

[25] Lee E.C., Jung H., Kim D. (2011). New finger biometric method using near infrared imaging. Sensors.

[26] Yang G.P., Xi X.M., Yin Y.L. (2012). Finger vein recognition based on a personalized best bit map. Sensors.

[27] Lee H.C., Kang B.J., Lee E.C., Park K.R. (2010). Finger vein recognition using weighted local binary pattern code based on a support vector machine. J. Zheijiang Univ. Sci..

[28] Bosch A., Zisserman A., Munoz X. (2007). Representing Shape with a Spatial Pyramid Kernel.

[29] Tibshirani R. (1996). Regression shrinkage and selection via the LASSO. J. R. Stat. Soc. B.

[30] Lazebnik S., Schmid C., Ponce J. Beyond Bags of Features: Spatial Pyramid Matching for Recognizing Natural Scene Categories.

[31] Ojala T., Pietikainen M., Harwood D. (1996). A comparative study of texture measures with classification based on feature distributions. PatternRecognit..

[32] Dalal N., Triggs B. (2005). Histograms of Oriented Gradients for Human Detection.

[33] Ng A.Y. Feature Selection, L1 *vs.* L2 Regularization, and Rotational Invariance.

[34] Liu J., Ji S., Ye J. (2009). SLEP: Sparse Learning with Efficient Projections.

